# Genetic Variants miR-126, miR-146a, miR-196a2, and miR-499 in Polycystic Ovary Syndrome

**DOI:** 10.3389/bjbs.2021.10209

**Published:** 2022-01-07

**Authors:** R. Li, Y. Yu, S. O. Jaafar, B. Baghchi, M. Farsimadan, I. Arabipour, H. Vaziri

**Affiliations:** ^1^ Department of Abdominal and Pelvic Medical Oncology, Huangshi Central Hospital, Affiliated Hospital of Hubei Polytechnic University, Huangshi, China; ^2^ Department of Obstetrics and Gynecology, Nanchong Central Hospital, Nanchong, China; ^3^ Department of Obstetrics and Gynecology, College of Medicine, Hawler Medical University, Erbil, Iraq; ^4^ Department of Emergency Medicine, Faculty of Medicine, Kermanshah University of Medical Sciences, Kermanshah, Iran; ^5^ Department of Biology, Faculty of Sciences, University of Guilan, Rasht, Iran; ^6^ Department of Biotechnology, Science and Research Branch, Islamic Azad University, Tehran, Iran

**Keywords:** microRNA, polycystic ovary syndrome, polymorphism, genetic, variants

## Abstract

**Introduction:** Alterations in certain microRNAs (miRNAs) and their target genes have reported in polycystic ovary syndrome (PCOS) and other disease of the female reproductive system, and so may be potential biomarkers. We hypothesised alterations in the prevalence of four miRNAs single nucleotide polymorphism (SNP) variants miR-126 rs4636297, miR-146a rs2910164, miR-196a2 rs11614913, and miR-499 rs3746444 in women with PCOS in comparison to healthy controls.

**Methods:** SNPs in the four miRNAs were determined in 385 patients and 385 controls by standard RT-PCR techniques.

**Results:** SNPs in miR-126 and miR-246a were significant linked with PCOS under the allelic, dominant, co-dominant, and recessive models (all *p* ≤ 0.01). The SNP in miR-499 was linked to PCOS in allelic (T, *p* = 0.002), dominant (*p* = 0.035) and recessive (*p* = 0.003) models. The SNPs -196a was significant linked to PCOS only in the recessive model (*p* = 0.037). Combining these SNPs in miR-499, mi146a, miR-196a and miR-126 respectively into allele haplotypes found highly significant odds ratios (95% CI) of 0.40 (0.29–0.54) (*p* < 0.001) for the C-G-C-G haplotype, and 0.46 (0.30–0.70) (*p* = 0.002) for the C-C-C-A haplotype (*p* = 0.002) for PCOS.

**Conclusion:** Single SNPs and haplotype combinations in certain SNPs in miR-126, miR-146a, miR-196a2 and miR-499 are strongly linked to PCOS, and so may be useful predictors of this condition.

## Introduction

Around 15% of couples suffer from infertility and about 37% of all infertility cases are due to female infertility [1–3]. Polycystic ovary syndrome (PCOS), a common cause of infertility, affects 6–10% of women of reproductive age. Patients with this syndrome manifest different symptoms including, menstrual irregularity, overweight, acne, and hair growth on their face and body [4]. The etiology of this disease is not well established, although genetic, endocrine, and physiological factors to significantly be contributing to is the pathogenesis [1–4].

MicroRNA (miRNAs) and their target gene in PCOS development in different studies and have reported various miRNAs to be potential biomarkers and a new approach for the diagnosis of PCOS. miRNA are small noncoding RNAs with 21–25 nucleotide length [5] that can repress mRNA translation by binding to their 3′-UTR or lead to the degradation of specific mRNAs and with this way play critical roles in the key biological function [6]; hence, the identification of human disease-linked miRNAs would effectively help to understand the pathogenesis of different disorders.

Single nucleotide polymorphism (SNPs) in microRNA genes have shown to considerably interfere with cellular functions mediated by these miRNAs; hence, disrupting the regulation and generation of these controlling elements in the cell [7]. Although many studies have investigated the association of SNPs with various diseases [8–11], there are few clinical studies on the effects of miRNAs variants in female reproductive disorders such as PCOS [12–16]. Accordingly, we hypothesised links between four miRNA variants miR-126 rs4636297, miR-146a rs2910164, miR-196a2 rs11614913, and miR-499 rs3746444 and PCOS, intending to further clarify the significance of miRNA variants in reproductive diseases.

## Methods

We tested our hypothesis in 385 cases with PCOS and 385 healthy controls. The criteria for the diagnosis of the PCOS patients was based on two of the three following features, according to Rotterdam 2003 criteria: (I) oligomenorrhea (menstrual period length greater than 35 days) or amenorrhea (menstrual period absent for 6 months), (II) clinical and/or biochemical signs of hyperandrogenism, (III) polycystic ovaries morphology as seen on ultrasound (at least one ovary contained >12 follicles measuring 2–9 mm in diameter and/or increased ovarian volume of at least 10 ml). Patients having other reasons of hyperandrogenism or menstrual irregularity such as Cushing’s syndrome, prolactinoma, and congenital adrenal hyperplasia were excluded. Pregnancy cases and women with the first postpartum year were not included in our study. Patients who received any hormonal medicine 3 months prior to this research were not included. The population of healthy controls included in this study were healthy women with the regular menstrual cycle (menstrual period up to 7 days and menstrual cycle of 21–35 days), normal androgen rates, no symptoms of hirsutism, and no history of PCOS. The study has been approved by the high graduate committees of the Faculty of Medicine/University of Guilan, Rasht, Iran. The informed consent documents were obtained from all subjects prior to sampling.

Blood samples were collected and genomic DNA was extracted from white blood cells with the Blood and Cell Culture DNA kit (Sinacolon, Tehran, Iran) according to the manufacturer’s protocol. Samples were first lysed and proteins simultaneously denatured in the lysis buffer. Protease or proteinase K was then added and after the incubation period, lysates were loaded onto the Genomic-tip to let the DNA bind to the column while other cell constituents pass through. Following a wash step to remove any remaining contaminants, pure, high-molecular-weight DNA was then eluted and precipitated with isopropanol. DNAs were analyzed by electrophoresis on 2% agarose gels with ethidium bromide staining.

The polymerase chain reaction-restriction fragment length polymorphism (PCR-RFLP) was used to determine four polymorphisms of miRNAs, i.e. miR-146a G>C, miR-126 G>A, miR-196a2 C>T and miR-499 T>C. A summary of the information of SNPs is shown in Table 1. The PCR conditions started with an initial denaturation at 94°C for 5 min, 35 cycles of denaturation of molecules at 94°C for 1 min, annealing at 59°C for rs4636297, 60°C for rs11614913, 61°C for rs2910164, and 57°C for rs3746444 for 1 min, and the products were under extension at 72°C for 40se, and a final extension at 4°C for 5 min in Biorad Thermocycler. The PCR products were separated on agarose gel and visualized by ethidium bromide staining. Total RNA was isolated using a commercial kit (Sinacoln, Tehran, Iran) following the manufacturer’s protocol. One microgram of total RNA was reverse transcribed into cDNA utilizing reverse transcription kits (Sinacoln). After cDNA conversion, quantitative PCR was done using SinaSYBR Master Mix (Sinaclon) and real-time PCR machine (Applied Biosystems, Waltham, Mass, United States). We used U6 snRNA as the reference gene. The relative expression for each candidate miRNA within each group was then calculated using the equation 2^−ΔΔCT^.

**TABLE 1:** General information of selected SNPs.

SNP	Position	Type of variant	Change	Primer sequences	Enzyme	Products length (bp)
miR-126 rs4636297	chr9:136670698	Downstream Transcript	NR_029695.1:n. A>G	CCC​GGA​GCC​TCA​TAT​CAG​C	HaeII	285,192,93
				GCT​ATG​CCG​CCT​AAG​TAC​GTC		
miR-146a rs2910164	chr5:160485411	Non Coding Transcript	NR_029701.1:n.60C>G	CAT​GGG​TTG​TGT​CAG​TGT​CAG​AGC​T	SacI	147, 120, 27
				TGC​CTT​CTG​TCT​CCA​GTC​TTC​CAA		
miR-196a rs11614913	chr12:53991815	Non Coding Transcript	NR_029617.1:n.78C>T	CTT​ACC​CAC​CCA​GCA​ACC​C	HpyCH4III	360, 218, 142
				CCC​CAC​TCA​CAG​CTT​GTC​C		
miR-499 rs3746444	chr20:34990448	Non Coding Transcript	NR_039912.1:n.25T>G	GCC​CCT​TGT​CTC​TAT​TAG​CTG	Tsp451	416,232.184
				ACT​TTT​GCT​CTT​TCA​CTC​TCA​T		

All statistical analyses were done using both SNPAlyze software (ver.8.1, Dynacom, Japan) and SPSS (ver.22). Allele and genotype frequencies of the SNPs among control and case groups were compared and checked using Pearson χ^2^ statistic. Moreover, deviations from Hardy–Weinberg equilibrium (HWE) were tested using a χ^2^ goodness-of-fit test. Analyses were also performed assuming recessive, codominant, and dominant models of inheritance and crude odds ratio (OR), their 95% CI ranges. Haplotype analyses were done using rs3746444, rs2910164, rs11614913, and rs4636297, variants for all study samples according to the maximum-likelihood method with an expectation–maximization algorithm. The significance level of the statistical tests was selected to be < 0.05.

## Results

Cases and controls were matched for age, BMI, FSH and successful pregnancies, although the cases had higher LH, history of miscarriage and of premature delivery (Table 2). The frequencies of SNP alleles in both groups are in accordance with the Hardy–Weinberg equilibrium (Table 3). As regards miR-126 rs4636297, cases were significantly more likely to carry an A allele, and co-dominant GA and recessive AA genotypes, but less likely to carry the dominant GG genotype. Similarly, those carrying the C allele, or the GC co-dominant or CC recessive genotypes of miR-146a rs29101164 were more likely to have PCOS, but those with the GG genotype were more likely to be free of this disease. The recessive TT genotype in miR196a rs11614913 was marginally more common in those with PCOS. The T allele in miR-449 rs3746444 was more common in PCOS, as was the dominant TT genotype, but the CC allele was more common in the controls.

**TABLE 2:** The demographic and biochemical characteristics of PCOS women and controls.

Variables	Patients (385)	Controls (385)	*p*-value
Age (years)	28.1 ± 0.31	28.6 ± 0.20	0.28
BMI (kg/m^2^)	26.6 ± 5.7	26.1 ± 4.8	0.13
FSH (mIU/ml)	6.5 ± 2.2	5.9 ± 2.5	0.22
LH (mIU/ml)	15.6 ± 4.1	5.74 ± 1.2	**<0.001**
At least one successful pregnancy (n, %)	181/202 (89)	242/258 (93)	0.10
Only one successful pregnancy (n, %)	50 (24)	58 (27.6)	0.39
More than one successful pregnancy (n, %)	131 (72.3)	184 (76)	0.38
History of miscarriage (n, %)	39 (19)	26 (9)	**<0.001**
History of premature deliveries (n, %)	25 (12.3)	11 (4)	**<0.001**

BMI, body mass index; FSH, follicle stimulating hormone; LH, luteinizing hormone. Data presented as mean with SD or as n (%).

**TABLE 3:** Allele and genotype distribution frequencies of every four SNPs in the case and control groups.

SNP	Model	Control	Case	OR (95%CI)	Adjusted *p*-value
miR-126 rs4636297	**Allele G**	607 (78.8)	537 (69.7)		
	**Allele A**	163 (21.2)	233 (30.3)	1.61 (1.28–2.03)	**0.0001**
	**Co-dominant (GA v GG + AA)**	135 (35.8)	169 (43.8)	1.69 (1.26–2.26)	**0.0004**
	**Dominant (GG v GA + AA)**	236 (61.2)	184 (47.7)	0.57 (0.43–0.77)	**0.0004**
	**Recessive (AA v GG + GA)**	14 (3)	32 (8)	2.40 (1.26–4.57)	**0.010**
		P^b^ = 0.06	P^a^ = 0.32		
miR-146a rs2910164	**Allele G**	607 (78.8)	494 (64)		
	**Allele C**	163 (21.2)	276 (36)	2.08 (1.65–2.61)	**<0.001**
	**Co-dominant (GC v GG + CC)**	119 (31)	168 (43.6)	1.73 (1.28–2.32)	**<0.001**
	**Dominant (GG v GC + CC)**	244 (63.3)	163 (42.3)	0.42 (0.31–0.56)	**<0.001**
	**Recessive (CC v GG + GC**	22 (6)	54 (14)	2.69 (1.60–4.51)	**0.003**
		P^b^ = 0.14	P^a^ = 0.31		
miR-196a rs11614913	**Allele C**	496 (64.4)	462 (60)		
	**Allele T**	274 (35.6)	308 (40)	1.21 (0.98–1.49)	0.074
	**Co-dominant (CT v CC + TT)**	178 (46.2)	168 (42.3)	0.79 (0.59–1.05)	0.126
	**Dominant (CC v CT + TT)**	159 (41.2)	147 (38.6)	0.87 (0.65–1.16)	0.352
	**Recessive (TT v CC + CT)**	48 (12.5)	70 (18)	1.56 (1.04–2.32)	**0.037**
		P^b^ = 0.86	P^a^ = 0.07		
miR-499 rs3746444	**Allele T**	398 (51.6)	463 (60)		
	**Allele C**	372 (48.4)	307 (40)	1.95 (1.13–2.86)	**0.002**
	**Co-dominant (TC v TT + CC)**	188 (48.8)	195 (50.6)	1.07 (0.81–1.42)	0.61
	**Dominant (TT v TC + CC)**	105 (27.2)	134 (34.8)	1.81 (1.31–2.45)	**0.035**
	**Recessive (CC v TT + TC)**	92 (23.8)	56 (14.5)	0.54 (0.37–0.78)	**0.002**
		P^b^ = 0.66	P^a^ = 0.26		

a HWE *p*-value for cases.

b HWE *p*-value for controls.

Bold values show the significant values. *p* < 0.05 was considered significant.

To further assess potential roles for these SNPs, we combined leading alleles into haplotypes. The combination of the C-G-C-G alleles in rs3746666, rs2910164, rs11614913 and rs4636297 respectively was markedly less common in those with PCOS, as was the C-C-C-A haplotypes. Of modest significant were the haplotypes T-C-T-G, T-C-C-A, and T-C-C-A, all of which were less likely to be present in the cases (Table 4).

**TABLE 4:** Distribution of haplotype blocks in PCOS patients and controls.

rs3746444	rs2910164	rs11614913	rs4636297	Control frequency %	Case frequency %	OR (95% CI)	Adjusted *p*-value
C	**G**	**C**	**G**	19.5	8.0	0.40 (0.29–0.54)	**<0.001**
C	**C**	**C**	**A**	4.5	1.1	0.46 (0.30–0.70)	**0.002**
T	**C**	**T**	**G**	5.9	1.2	0.21 (0.06–0.74)	**0.01**
T	**C**	**C**	**A**	3.7	2.0	0.47 (0.28–0.85)	**0.01**
T	**C**	**C**	**G**	9.1	5.5	0.52 (0.35–0.78)	**0.01**
T	**C**	**T**	**A**	2.0	1.3	0.46 (0.19–1.11)	0.06
C	**G**	**T**	**A**	4.2	1.7	0.76 (0.40–1.18)	0.11
T	**G**	**C**	**A**	6.9	4.7	0.61 (0.35–1.04)	0.10
C	**C**	**T**	**A**	1.3	0.0	0.83 (0.25–1.15)	0.76
T	**G**	**T**	**G**	11.9	12.9	1.09 (0.85–1.40)	0.54
T	**G**	**T**	**A**	2.4	4.2	1.77 (0.68–2.92)	0.07
T	**G**	**C**	**G**	20.6	17.0	0.75 (0.58–1.07)	0.06
C	**G**	**T**	**G**	10.3	7.5	0.70 (0.42–1.06)	0.11
C	**C**	**C**	**G**	5.4	5.2	0.67 (0.33–1.38)	0.37
C	**G**	**C**	**A**	5.0	5.6	1.45 (0.72–2.22)	0.10

Bold values show the significant values. *p* < 0.05 was considered significant.

In PCOS patients, miR-126 and miR-146a serum levels were significantly lower than controls [(0.85 ± 0.1 vs. 0.45 ± 0.1) (*p* = 0.02) and (0.82 ± 0.2 vs. 0.36 ± 0.1) (*p* < 0.001)], while no significant differences were observed for miR-196a and miR-499 levels in cases compared with controls (0.73 ± 0.2 vs. 0.52 ± 0.1) (*p* = 0.23); (0.65 ± 0.1 vs. 0.55 ± 0.2); (*p* = 0.69).

In miR-146a, CC genotype showed significantly lower serum level, followed by GC then GG. A borderline association was found when comparing the expression level of miR-146a GC (0.38 ± 0.2 vs 0.69 ± 0.1) (*p* = 0.05), but the CC difference (0.33 ± 0.2 vs. 0.99 ± 0.26) was significant (*p* > 0.001) in patients and controls; however, no difference was observed for miR-146a serum level in GG model (0.5 ± 0.15 vs 0.62 ± 0.1) (*p* = 0.31). For miR-126 genotypes, the highest expression of serum miR-126 was detected in CC genotype, followed by TT and lastly CT genotype in both cases and control. The difference was highly statistically significant when comparing the serum levels of miR-126 CC in cases and controls [CC (0.92 ± 0.1 vs. 0.23 ± 0.1) (*p* < 0.001), but not in CT (0.65 ± 0.2 vs.0.5 ± 0.15) (*p* = 0.52) or TT (0.88 ± 0.1 vs. 0.75 ± 0.2) (*p* = 0.68)]. Serum miR-196a levels did not associated with any genotype of rs11614913 C>T polymorphism between the patients and healthy group (CC (0.82 ± 0.25 vs. 0.51 ± 0.4) (*p* = 0.7); CT (0.71 ± 0.2 vs. 0.48 ± 0.1) (*p* = 0.49); TT (0.6 ± 0.1 vs. 0.55 ± 0.15) (*p* = 0.36) respectively) and expression levels of miR-499 was only significant in recessive model in PCOS women compared to controls (0.65 ± 0.3 vs 0.2 ± 0.1) (*p* = 0.01).

## Discussion

In this study, we investigated the effect of four SNPs in PCOS women in comparison to healthy controls and found a significant association of miR-126 rs4636297, miR-164a, rs2910164, and miR-499 rs3746444 with PCOS. We suggest that these miRNAs could contribute to the pathogenesis of PCOS.

Our data add to the general literature of this group of molecules, which also include miR-21, miR-27b, miR-222, miR-146a, miR-486-5p, and miR-103 [12–16]. Some of these changes may have profound pathophysiological implications. For example, Zhao et al. showed the importance of miR-323-3p in cell proliferation and apoptosis by showing the role of this miRNA in regulation of PDCD4, a tumor suppresser gene, in PCOS patients [16]. Likewise, the important role of miR-17-5p in cell proliferation, regulation of PETN (another tumor suppressor), and cell apoptosis of ovarian granulosa cells was indicated in another study [11]. Several studies have indicated the critical role of miRNAs SNPs in ovarian function, development, and disorders; however, only a few studies have been focused on the role of miRNAs variants in PCOS, as one of these significant disorders. Hosseini et al. in a study investigating the association of miR-146a and miR-222 polymorphisms with susceptibility to PCOS demonstrated the significant correlation of both SNPs with an increased risk of PCOS [17]. Similar to our results, Ebrahimi and others found a significantly higher rate of miR-146a variant incidence in cases compared to controls and considered miR-146a variation to lead to increased PCOS among their studied group [18].

The association of microRNA polymorphisms with recurrent spontaneous abortion (RSA) was frequently reported in different studies and showed a significant correlation of miR-146aC>G, miR-196a2T>C, and miR-499A>G with idiopathic recurrent spontaneous abortion [19–21]. In contrast, Fazil et al. failed to show any relationship between the increased rates of recurrent miscarriage and miR-196a2 gene variation; however, they considered miR-499 gene variant to be a predisposing factor [22]. A recent study that investigated the link between polymorphisms in miRNA-196a2 and miRNA-499 in maternal blood and the placentas of patients with preeclampsia reported miRNA-196a2 rs11614913 variant to be a protective factor for preeclampsia predisposition among the studied group [23].

Several studies have also confirmed the strong relationship of miRNA SNPs with increased risk of endometriosis incidence among women. A study of 157 endometriosis patients and 252 healthy women, mir-126 rs4636297 significantly differed between the two groups, the authors speculating a role in individual’s susceptibility to endometriosis and its severity [24]. Similarly, Farsimadan et al. in a very recent study investigating the association of miR-146a, miR-149 rs2292832, miR-196a2, and miR-499 variants with the higher rates of endometriosis, suggested the importance of these SNPs with disease severity [25]. In addition, Chang et al. indicated that genetic variants in miR196A2 (rs11614913) was strongly associated with endometriosis development and related clinical phenotypes, such as infertility [26]. The significant role of miR-146b and its variants in endometriosis further was supported in a recent study in China that reported miR-146b to have an essential role in regulating the process of endometriosis and its associated pain [27]. Others confirmed the important role of miR-126 in the development and progression of endometriosis [28–31]. The miR-499 rs3746444 had also a potential function in reducing the risk of endometrial cancer [32]. The significance of miR-196a2, miR-126, and miR-499 in the initiation and development of cervical cancer has been reported in different studies as well [33–37].

Our data have potential screening and clinical implications, identifying girls and young women who may be at risk of PCOS, being already present in older family members. For example, the miR-146 AA genotype brings >2.5-fold likelihood of PCOS, whilst the miR-146a CC genotypes brings a >2-fold increased likelihood, and although these data have an odds ratio >2, we concede these data are based on small numbers. We are also unable to speculate that these data represent risk ratios, which can only be determined in large prospective studies of at-risk populations. Similarly, the haplotype data also point to a value in these SNPs, where certain combinations are less frequent in PCOS, and accordingly speculate that the presence of these haplotypes are protective from this disease.

This work represents an advance in biomedical science because it shows a link between PCOS and certain miRNAs variants, prompting a marker role in reproductive biology.

## Summary Table

### What is Known About This Subject

• PCOS is a common disease of the female reproductive system of unclear aetiology
• miRNAs variants in miR-126 rs4636297, miR-146a rs2910164, miR-196a2 rs11614913, and miR-499 rs3746444 have been linked to various female reproductive disorders.

### What This Study Adds

• Certain SNPs in miR-126, miR-146a and miR-499 are very significant linked (*p* ≤ 0.002) with PCOS.
• Combining these various SNPs in miR-146a, miR-196a and miR-126 into allele haplotypes also found highly significant links with PCOS.

## Data Availability

The original contributions presented in the study are included in the article/supplementary material, further inquiries can be directed to the corresponding author.
